# Myoclonic Disorders

**DOI:** 10.3390/brainsci7080103

**Published:** 2017-08-14

**Authors:** Olaf Eberhardt, Helge Topka

**Affiliations:** Klinik für Neurologie, Klinikum Bogenhausen, Städt. Klinikum München GmbH, Englschalkinger Str. 77, 81925 München, Germany; olafeberhardt@web.de

**Keywords:** movement disorders, myoclonus, epilepsy, neurogenetics, encephalopathies

## Abstract

Few movement disorders seem to make a straightforward approach to diagnosis and treatment more difficult and frustrating than myoclonus, due to its plethora of causes and its variable classifications. Nevertheless, in recent years, exciting advances have been made in the elucidation of the pathophysiology and genetic basis of many disorders presenting with myoclonus. Here, we provide a review of all of the important types of myoclonus encountered in pediatric and adult neurology, with an emphasis on the recent developments that have led to a deeper understanding of this intriguing phenomenon. An up-to-date list of the genetic basis of all major myoclonic disorders is presented. Randomized studies are scarce in myoclonus therapy, but helpful pragmatic approaches at diagnosis as well as treatment have been recently suggested.

## 1. Introduction

Myoclonus is characterized by sudden, brief, shock-like involuntary movements, associated with bursts of muscular activity (positive myoclonus) or silencing of muscular activity (negative myoclonus) [1]. It may be present at rest, during voluntary movement (action-induced) or due to provoking stimuli such as sensory, visual, auditory or emotional cues. Myoclonus at rest is observed in epileptic disorders, spinal myoclonus, posthypoxic myoclonus or Creutzfeldt–Jakob disease, for example.

Typically, myoclonus presents as short (10–50 ms, rarely more than 100 ms), non-rhythmic jerks, often without any discernible pattern. Exceptions from this default description of myoclonus do exist. Exceptional long-lasting discharges, e.g., in Creutzfeldt–Jakob disease, have been named dystonic myoclonus [2]. These patterns have to be differentiated from longer-lasting spasms in tetanus or rabies, or from motor stereotypies in childhood. Rather rhythmic movements, e.g., appearing every 50–80 ms, can also be part of the spectrum of cortical myoclonus. Rhythmic myoclonus may show in epilepsia partialis continua, familial cortical myoclonic tremor, some cases of progressive myoclonic epilepsies, corticobasal degeneration, posthypoxic myoclonus or spinal segmental myoclonus. Rhythmic myoclonus can be mistaken for tremors. Some cases of cortical tremors have been identified as cortical myoclonus based on electrophysiology, for example [3]. In these instances, agonist and antagonist muscles are involved simultaneously; a feature that is rather rare in tremors.

The amplitude of myoclonus can vary considerably depending on subtype. The lightning-like, square-wave character of myoclonus helps in its differentiation from tremors (rhythmic oscillations), chorea (larger, random flowing movements), dystonia (geste antagonistique, burst duration > 100 ms, often sustained twisting posturing), tics (burst duration > 100 ms, may be suppressed temporarily) or fasciculations (single muscles, minimal movement effect) [4,5].

Myoclonus does not necessarily represent a pathological phenomenon. Physiologic myoclonus occurs during sleep transition or during sleep itself (hypnic jerks), for example. In many instances, myoclonus is just one of many symptoms of a complex neurodegenerative or epileptic disorder. What is more, a plethora of metabolic derangements and many types of medication can transiently cause myoclonus.

Data on the incidence or prevalence of myoclonus are scarce. The average annual incidence rate of pathologic and persistent myoclonus in one study was about 1 per 100,000 person-years and the lifetime prevalence of myoclonus was less than 10 cases per 100,000 population [6]. The rate increased with advancing age and was consistently higher in men. Symptomatic myoclonus was the most common type, followed by epileptic and essential myoclonus. Neurodegenerative and dementing diseases were the most common cause of symptomatic myoclonus (about 70%) [7].

Strategies of systematization have led to overlapping, not mutually exclusive classifications (Table 1). Clinical, etiological or neuroanatomical classifications have been suggested (Table 1). A separation between epileptic and non-epileptic myoclonus promises to be a welcome start, but proves difficult in clinical practice. The phenomena of positive and negative myoclonus add another layer of complexity.

## 2. The Anatomical Approach to Categorization

Following the subdivision of pyramidal, extrapyramidal and segmental myoclonus suggested by Halliday (1967) [8], the anatomical categorization has been refined in recent years based on electrodiagnostic studies. Most often, cortical, subcortical and segmental myoclonus form the basis of recent anatomical subdivisions, whereas some authors add cortical–subcortical myoclonus as a fourth category. Positive electrodiagnostic criteria mainly exist for cortical myoclonus. Thus, the finding of a back-averaged cortical negativity preceding the jerks or a giant somatosensory evoked response may point to a cortical myoclonus generator, while subcortical myoclonus lacks these features. On the other hand, enhanced long-loop muscular responses correlate with reflex myoclonus that may be found in cortical and subcortical (reticular) myoclonus alike.

We attempt to provide an anatomic classification of myoclonic disorders according to their presumed anatomic source of myoclonus. It must be emphasized, however, that in some disorders the empirical evidence underlying this classification is tenuous or ambiguous; for example, in prion diseases. Thus, this anatomic classification may be preliminary in some respect. Furthermore, some authors have advocated subcortical contributions to myoclonus even in disorders classically assumed to have a cortical origin [9]. Due to a lack of unequivocal data, it is not yet possible to reliably attribute the anatomic sources of myoclonus in metabolic-toxic, infectious or autoimmune disease.

### 2.1. Cortical Myoclonus

Cortical myoclonus generators are probably the most frequent neuroanatomical substrate of myoclonus [7], but not necessarily in isolation. Many conditions involving cortical myoclonus also present with epileptic seizures [10]. Consequently, one may find multifocal or generalized spike-waves in EEG, implying enhanced cortical excitability. Pre or post-central cortex and pyramidal neurons of layer III and V are hyperexcitable structures in these conditions, possibly related to GABA-ergic (Gamma-Aminobutyric Acid) mechanisms and altered cortico-thalamic connectivity [11,12]. In cortical myoclonus, short-interval intercortical inhibition is reduced, probably as a consequence of the GABA-ergic dysfunction of cortical inhibitory interneurons [13]. GABA-A-dependent intrahemispheric or interhemispheric disinhibition may both be suggested by transcranial magnetic stimulation, and there may be an increased corticomuscular coherence of high-frequency oscillations [14,15].

Cortical myoclonus is usually action-induced or sensitive to somatosensory, or occasionally to visual, stimuli or emotional cues. It typically presents with focal or multifocal arrhythmical jerks that often involve the face or distal (upper) extremities. The movements are often multifocal due to intra- and interhemispheric spread. They involve agonist and antagonist muscles simultaneously and show rostrocaudal recruitment. Compared to subcortical myoclonus, cortical myoclonus tends to be of shorter duration. Surface polymyography proves multifocal short duration bursts (20–70 ms). Rest, action and reflex myoclonus in a multifocal distribution can be simultaneously present. Negative myoclonus occurs frequently. When multifocal cortical myoclonus spreads to the opposite side of the body, homologous arm muscles are affected some 10–15 ms later, a latency difference that might be missed in an initial electrophysiologic evaluation and hence may lead to the incorrect identification of primarily generalized discharges [2].

Back-averaging employs electrode positions C3/C4 (according to the 10/20 EEG system) for the arms, or Cz for the legs, and correlates averaged responses with EMG (Electromyography) bursts used as a trigger. This technique has been in use since 1975. A focal, time-locked cortical discharge on EEG-EMG back-averaging that precedes the EMG activity can be found. This EEG discharge is usually smaller than focal epileptiform discharges. Typically, a biphasic, often small (5–20 µV) EEG transient (spike, polyspike) of 15–40 ms duration can be recorded in cortical myoclonus prior to the recorded movement response. Its latency is about 20 ms for arm muscles and about 30 ms for leg muscles [14,16]. Back-averaging of magnetoencephalogram (MEG) data may be even more sensitive in spike detection than classical EEG-EMG back-averaging [17] though technically more challenging.

Giant median nerve SEP (Somatosensory evoked potentials) responses (P27/N35) following a N20 response of normal size may be observed. Enlarged SEP late responses, often more than tenfold enlarged or larger than 10 µV, can thus support the diagnosis of cortical myoclonus, but are not always present. On the other hand, giant SEPs are non-specific and can also be found in cortical atrophy or dementia without myoclonus [18]. Pain SEP responses are of normal size, and responses in proprioception-related SEP are variable [19].

Long-loop muscular responses 40–45 ms (and 10–15 ms later on the non-stimulated side) after median nerve stimulation, called C-reflex, correspond to transcortical reflexes in cortical reflex myoclonus [18]. They rarely occur in resting muscle in normal subjects, may be more difficult to interpret during slight muscle activation, and are not entirely specific to myoclonic disorders [18]. Typical (though not undisputed) examples of cortical myoclonus include progressive myoclonic epilepsies, juvenile myoclonic epilepsies, some forms of posthypoxic myoclonus, Alzheimer’s disease, Creutzfeldt–Jakob disease, Parkinson’s disease, corticobasal syndrome, some metabolic encephalopathies, lithium-induced and other drug-induced disorders. The elucidation of the genetic basis of many of these disorders has made considerable advances. For familial adult myoclonic epilepsy, for example, five genetic loci have been defined ([20], see Table 2).

Some authors have described a cortical–subcortical myoclonus subtype that shows myocloni of up to 100 ms and may present with generalized seizures at rest. The existence of this category as a separate entity is dubious.

#### 2.1.1. Myoclonus in the Context of Epilepsy

Neuronal hyperexcitability or abnormal paroxysmal depolarizations in epilepsy might generate motor features including positive or negative myoclonus. Myoclonus can therefore be considered a fragment of epilepsy in many related disorders, either as focal/multifocal myoclonus representing fragments of partial epilepsy, or as a fragment of generalized epilepsy; e.g., in reticular reflex myoclonus or primary generalized epileptic myoclonus [21]. Epileptic cortical myoclonus can occur spontaneously, induced by movement or by somatosensory stimuli (action and reflex myoclonus, respectively). Myoclonus can be a seizure component, the only manifestation of seizure or one of several seizure types within an epileptic syndrome. In diseases such as absence epilepsies, myoclonus can be just a subtle feature.

Other epileptic motor features have to be differentiated. Jacksonian spread across the motor cortex is usually relatively slow, taking many seconds or even minutes. Motor partial seizures often involve the upper extremities but, unlike myoclonic seizures, also commonly the lower extremities, alone or together with the upper extremities.

As Dawson observed in 1946 after carefully recording serial myoclonic seizures, “no case of jerking occurring synchronously with the spikes in the EEG has been seen when they have not appeared in the precentral leads … no action potential or detectable muscular jerk has been found to accompany a single isolated spike in the EEG [22]. Two spikes are usually accompanied by a minimal action potential in flexor muscles alone, whilst when a longer burst of spikes occurs action potentials most commonly appear first in the flexor muscles, follow in the extensor muscles and recruit steadily up to the end of the spike burst, then cease suddenly” [22].

##### Progressive Myoclonic Epilepsies

There are several distinctive groups of epileptic disorders associated with myoclonus. The progressive myoclonic epilepsies (PME) are a group of hereditary, mostly autosomal recessive disorders with progressive neurologic decline, the most important of which represent Unverricht–Lundborg Disease (EPM1), Lafora disease (EPM2), the genetically heterogeneous neuronal ceroid lipofuscinoses, myoclonus epilepsy and ragged-red fibers (MERRF) and Gaucher disease type 3 [23,24] (see Table 2). Rarer examples include autosomal dominant dentatorubral-pallidoluysian atrophy (DRPLA). Only MERRF has a mitochondrial inheritance (Table 2). Depending on phenotypic presentation, some of these disorders rather merit the term progressive myoclonic ataxia [25].

The emergence of myoclonus and epilepsy in adolescence should direct attention to progressive myoclonic epilepsies, juvenile myoclonic epilepsy (JME), GM2 gangliosidosis (Sandhoff, Tay–Sachs), Niemann–Pick disease type C or other respiratory chain defects than MERFF (Table 2). There may be accompanying features, such as cognitive decline, visual loss or cerebellar ataxia, that lead to the diagnosis of PME. MERRF, sialidosis or ceroid lipofuscinosis can also have an adult onset. Myoclonus in ceroid lipofuscinosis can appear as late as in the fourth decade [26,27].

In progressive myoclonic epilepsies, arrhythmic, asynchronous and asymmetric myoclonus shows focal or segmental distribution, most prominent in the face and distal extremities. Myoclonus is frequently precipitated by posture, action or stimuli, such as light, sound, or touch. Small-amplitude jerks do not show a time-locked appearance with respect to EEG discharges, while some large-amplitude jerks may correlate with generalized spike-wave bursts [28]. Sialidosis shows intention and action myoclonus. In Unverricht–Lundborg disease and some cases of neuronal ceroid lipofuscinoses there can be marked photosensitivity. Apart from generalized tonic–clonic seizures there may be absences, tonic or focal seizures [23,24].

Therapeutically, valproic acid, clonazepam, piracetam, phenobarbital, levetiracetam, piracetam, topiramate and zonisamide have shown some effect, often used in combination. Emergency treatment may include intravenous diazepam, lorazepam, clonazepam, midazolam, valproic acid, and levetiracetam. Lamotrigine should be avoided as it may worsen cortical myoclonus. Similarly, phenytoin, carbamazepine, oxcarbazepine, gabapentin and pregabalin should generally not be used for treatment, and in mitochondrial disease valproic acid, zonisamide and topiramate should be avoided [24,29,30].

##### Juvenile Myoclonic Epilepsy

On the one hand, juvenile myoclonic epilepsy (JME, Table 2) usually starting in adolescence is considered a prototypical generalized epilepsy syndrome. On the other hand, imaging findings from PET and MRS (Magnetic Resonance Spectroscopy) have led to the suggestion that JME is a frontal lobe variant of a frontocortical–subcortical network epilepsy involving the thalamus, rather than a generalized epilepsy syndrome [31]. Characteristically, bilateral myoclonic seizures involving the upper body occur at full consciousness soon after awakening or sudden arousal, while most patients also present with generalized tonic–clonic seizures and some patients with absence seizures.

A small-amplitude twitching in various muscles sometimes seen in these patients has been called minipolymyoclonus. Some patients exhibit relatively slow arm movements lasting up to 1 s with slow relaxation [28].

Myoclonic jerks are associated with fast, rhythmic, generalized polyspike waves with frontal predominance. Jerk-locked averaging techniques have suggested a cortical origin of the myoclonic jerks, and transcranial magnetic stimulation studies provided evidence of reduced intracortical inhibition due to impaired GABA-A mediated mechanisms [32]. Photosensitivity in EEG is common (30%). Interictal EEG shows diffuse or generalized spike waves and polyspike waves. Up to a third of patients may also show focal EEG abnormalities [26].

Antiepileptic drugs such as valproic acid achieve seizure remission in two thirds of patients [33]. Myoclonic seizures may disappear or diminish in the fourth decade of life [34]. 15–30% of patients are later able to discontinue medication without seizure recurrence [34,35].

##### Other Rare Myoclonic Epilepsies

Myoclonic epilepsy in infancy is a self-limited disorder responsive to valproic acid [36]. Rare cases of cortical myoclonus and epilepsy (BAFME2, see Table 2) involve mutations of the α2B-adrenergic receptor that stimulate calcium signaling [37]. Familial cortical myoclonic tremor is a cortical reflex myoclonus resembling focal epileptic seizures, but lacking epileptic discharges in EEG [38]. Early-onset myoclonic epilepsy in glucose transporter 1 deficiency is responsive to high-dose steroids or ketogenic diet [39].

#### 2.1.2. Neurodegenerative and Mitochondrial Disorders

##### Wilson’s Disease

In rare cases (3%), Wilson’s disease, an autosomal recessive disorder characterized by the accumulation of hepatic copper and caused by homozygous or compound heterozygous mutation in the ATP7B gene on chromosome 13q14 (Table 2), presents with myoclonus. Cortical multifocal myoclonus has been reported. Positive or negative myoclonus might also result from severe hepatic dysfunction in Wilson’s disease. More characteristically, patients are afflicted by parkinsonism, dystonia, cerebellar ataxia or chorea [40]. In one large case series, about 8% of Wilson’s patients suffered from seizures, but only exceptionally from pure myoclonic seizures [41].

##### Prion Diseases

Creutzfeldt–Jakob’s disease (CJD) occurs as an inherited (15%), acquired or sporadic disease. Hereditary (autosomal dominant) forms show various coding mutations in the prion protein gene (PRNP) gene (Table 2). The onset of disease is earlier in familial cases. In sporadic cases, met129 homozygosity is a highly-enriched risk allele. Cumulatively, about 80% of patients each show gait disorders, myoclonus and cerebellar ataxia [42]. CJD may present with rest or action myoclonus; later in the disease also with stimulus-induced, pseudo-rhythmic myoclonus. Binelli et al. [43] categorized myoclonic jerks as periodic, rhythmic or irregular myoclonus and noted the presence of negative myoclonus in almost 20%. Periodic sharp-wave complexes were present in 98%, but were time-locked with EMG bursts only in cases with periodic myoclonus. Jerk-locked back-averaging did not confirm cortical myoclonus in many cases [43], as EEG transients tended to precede motor burst onset by more than 50 ms, which is unusual for a cortical generator [44].

Fatal familial insomnia is an allelic, hereditary prion disease due to mutation of the prion protein gene at codon 178, and causes action myoclonus, insomnia, ataxia and dementia [45] (Table 2). In another allelic prion disorder, Gerstmann–Sträussler–Scheinker disease (Table 2), myoclonus is much less common.

##### Parkinsonian Syndromes

In 5% of treated, non-demented Parkinson’s disease patients, a small-amplitude, multifocal cortical myoclonus (polyminimyoclonus) with a time-locked premovement potential has been reported [46,47]. There was no reflex myoclonus, no giant SEPs or changes in long loop reflexes. Corticomuscular coherence in the 12–30 Hz band was increased [48]. More frequently, cortical myoclonus is found in patients with mutations or multiplications of the α-synuclein gene [49] (Table 2).

Similarly, postural and action myoclonus of the hand and fingers may be seen in 30% of cases of multisystem atrophy, but with unremarkable SEP and back-averaging [50]. Multifocal action myoclonus that may be observed in 15%–30% of dementia with Lewy bodies displays similar characteristics: positive back-averaging, but no reflex myoclonus, giant SEPs or altered long loop reflex responses [51].

Patients suffering from corticobasal degeneration characteristically display a unilateral, often quite rhythmic action myoclonus which may also prove stimulus-sensitive. A majority of patients (55%) display this type of myoclonus during the course of the disease [52], but only 15% at first presentation [53]. They show enhanced long-loop responses but no giant SEPs and no preceding EEG spikes [54]. Myoclonus often proves responsive to clonazepam [52].

##### Alzheimer’s Disease

In Alzheimer’s disease, myoclonus may be an early feature in presenilin 1 mutation carriers (Table 2) and a frequent late feature in sporadic disease. For example, in one series of 72 patients the prevalence of myoclonus increased from 7% to 39% in the course of the disease [55]. Other studies with pathological confirmation of diagnosis reported prevalences of myoclonus between 10% and 55% [56,57]. Almost 30 presenilin 1 mutations, but not presenilin 2 or APP mutations, have been associated with the emergence of myoclonus [58]. Some cases with clinically diagnosed corticobasal syndrome and prominent myoclonus may in fact have underlying Alzheimer’s pathology [25].

Jerk-locked back-averaging can detect a contralateral negative EEG potential preceding the jerks, confirming its cortical origin in some patients [59,60], but a temporal correlation of myoclonus and seizure has not been found by others [56].

##### Huntington’s Disease

In juvenile Huntington’s disease (Table 2), cortical generators of myoclonus were identified by jerk-locked back-averaging, reduced intracortical inhibition and enhanced long-latency reflexes, but without giant SEPs [61]. Huntington’s disease phenocopies with myoclonus and sometimes dementia can be caused by C9orf72 hexanucleotide repeat expansions, dentatorubral-pallidoluysian atrophy (DRPLA) or mitochondrial disease [62,63].

##### Mitochondrial Disease

In a large Italian database, myoclonus has been recorded as a feature of mitochondrial disease in less than 4% of patients, and in only 25% of them at disease onset [64]. MERRF (Table 2) is a mitochondrial syndrome characterized by generalized seizures, myoclonus, and ataxia, often associated with the mitochondrial DNA point mutation 8344A > G. Myoclonus is present in 20% of patients harboring the 8344A > G mutation [64]. Ragged red fibers in muscle biopsies and normal brain MRIs are characteristic for carriers of this mutation. Myoclonus may also be found in MELAS (Mitochondrial encephalopathy, lactate acidosis and stroke-like episodes), Leigh syndrome, Alpers syndrome, Leber hereditary optic neuropathy (LHON) or POLG (Polymerase Gamma) spectrum (ataxia, polyneuropathy, ophthalmoplegia), but often outside the context of a classical mitochondrial syndrome [65]. In mitochondrial patients with myoclonus, seizures are a frequent (about 60%) associated symptom. Ataxia, hearing loss and cognitive impairment affect half of the patients, and ophthalmoparesis or neuropathy about 25% of patients [64]. Giant SEPs have been detected in only 2 of 9 examined cases.

##### Spinocerebellar Ataxia

Among the spinocerebellar ataxias, SCA2 is noteworthy as the subtype with the strongest association with myoclonus (Table 2). It may also be a feature in SCA6, 8, 13, 14 and 19 [10]. Myoclonus is an unusual feature in Friedreich ataxia.

#### 2.1.3. Cerebrovascular Disease with Probable Cortical Sources

Myoclonus can be a rare feature in patients with symptomatic occlusive disease of the internal carotid artery or middle cerebral artery with depleted hemodynamic reserve and has become known as limb-shaking transient ischemic attack [66]. Its epileptic nature has not been clearly defined, as a focal slowing seems to be the predominant EEG finding. Reperfusion stopped the symptoms in several reported cases; in one well-documented case, there was a 20 s delay from reperfusion to symptom relief [67]. Sometimes, epilepsy after pericentral stroke manifests as negative myoclonus that responds to anticonvulsant drugs [68].

#### 2.1.4. Acute Posthypoxic Myoclonus

Two main forms of posthypoxic myoclonus have been described, although sometimes in the same patient at different time-points. Acute posthypoxic myoclonus appears in the first one to three days after prolonged circulatory arrest. Multifocal or generalized jerks occur spontaneously or are stimulus-sensitive, often only as a temporary phenomenon [69]. Most frequently, burst suppression, spike waves or status epilepticus characterize the post-hypoxic EEG with myoclonus, although alpha coma or diffuse slowing may also be observed. These EEG features show variable temporal association with motor phenomena. As enlarged SEPs are rarely observed in post-hypoxic myoclonus, subcortical rather than cortical myoclonus generators have been suggested [69].

Following therapeutic hypothermia after cardiac arrest, 18% of patients develop myoclonus, but just about half of them (55%) with epileptiform activity in electroencephalograms [70]. Still, favorable outcomes may be observed in up to 9% of survivors, but in only 2% with epileptiform activity in electroencephalograms [70]. Earlier studies have reported a 0–7% chance of survival with myoclonus [71], and no surviving patients with status myoclonicus, but in many cases self-fulfilling decisions to limit therapy might have been influenced by prior negative experiences with this electroclinical constellation.

A rare variant has been described as early bilateral flexor postanoxic myoclonus, showing sudden trunk flexion, sometimes stimulus-induced or also involving the head or extremities. EEG studies show burst-suppression patterns, with muscle bursts coinciding with flexion spasms. An extremely poor prognosis is likely in these cases [28].

### 2.2. Subcortical Myoclonus

Subcortical myoclonus lacks back-averaged cortical potentials or enlarged somatosensory evoked responses. Long latency reflex alterations may occur, however. The category of subcortical myoclonus includes essential myoclonus, myoclonus-dystonia, reticular reflex myoclonus, startle syndromes, Creutzfeldt–Jakob disease and subacute sclerosing panencephalitis (SSPE) [44]. Myoclonic bursts variably last for 25–300 ms. Examples of subcortical myoclonus include some metabolic encephalopathies, some forms of posthypoxic myoclonus, progressive myoclonic epilepsies, myoclonus-dystonia or opsoclonus-myoclonus syndrome.

Reticular reflex myoclonus (brainstem reticular myoclonus) may present with generalized, synchronized jerks of short duration that are most pronounced in axial and proximal flexor muscles. Jerks sometimes also occur spontaneously [5]. Occasionally there are jerks limited to one region of the body [21]. This type of myoclonus is typically sensitive to multisensory stimuli and may be elicited by voluntary movements or sensory stimulation. Generalized spikes show no constant temporal association with myoclonic jerks. Long-loop reflex responses may be enhanced but no enlarged SEP is found. A myoclonus generator near the medulla has been postulated, as muscles supplied by the accessory nerve may show the earliest activation. The anatomically related but otherwise dissimilar exaggerated startle response involves bilaterally synchronous shock-like movements to sudden intense stimuli. This response habituates over time. Startle responses are characterized by eye closure, raising of the bent arms over the head and flexion of neck trunk and proximal joints [72]. Burst duration is about 50–400 ms. Adults may have a wide-based gait without ataxia and an exaggerated head retraction reflex [73]. Startle responses may be part of hyperekplexia syndromes (hereditary or sporadic) starting in childhood or puberty, or be a symptom of startle-induced epilepsy, various brainstem lesions or neuropsychiatric disease. Various mutations causing hereditary startle disease (hyperekplexia) cluster around glycine effector mechanisms; i.e., the glycine transporter or glycine receptor function (Table 2). It is assumed that the bulbopontine reticular formation mediates these reactions [72]. Clonazepam is often effective.

#### 2.2.1. Myoclonus Dystonia

Autosomal dominant myoclonus dystonia (Table 2) is an important differential diagnosis in young patients with prominent, slowly evolving myoclonus, as dystonic features can be subtle. Symptom onset can vary between 1 and 80 years, although most cases become symptomatic until early adolescence. Symptoms tend to plateau in adulthood. Depending on inclusion criteria, in genetic studies 20–80% were found to carry sarcoglycan-ε (SGCE) mutation [74,75,76,77]. Maternal imprinting prevents disease expression in 95–100% of children with a pathogenic maternal sarcoglycan-ε variant. Symptoms tend to plateau in adulthood. Myoclonus with long burst durations and dystonia predominantly involve neck and upper limbs [78]. Nevertheless, more to one third of patients initially show lower limb-predominant dystonia, although rarely lower-limb myoclonus [77]. Two thirds suffer from psychiatric co-morbidity, such as phobias, alcohol dependence or obsessive-compulsive disorders [79]. Increased alcohol consumption may be related to its relieving effect on myoclonus in some cases [80]. Typically, both dystonia and myoclonus improve with antagonistic gestures or postures.

In other patients with a similar clinical phenotype, vitamin E deficiency, dopa-responsive dystonia due to mutations in tyrosine hydrolase (Segawa syndrome) or guanosine triphosphate cyclohydrolase (GTP-CH), or uniparental disomy of chromosome 7 have been described [81]. Cerebrotendinous xanthomatosis may present with subcortical distal myoclonus and mild dystonia of the upper limbs, potentially responsive to chenodeoxycholic acid. Rare mutations in the D2 dopamine receptor [82] were obviously a coincidental non-pathogenic finding. SGCE-mutation-negative patients may also display truncal dystonia, tics or tremor unusual for SGCE mutations [77,78]. In a fMRI study, cerebellar dysfunction distinguished SGCE mutation-positive and negative patients [83]. Unilateral myoclonus dystonia may be due to thalamic lesions.

Cortical excitability and intracortical inhibition has been found to be normal or less profoundly disturbed than in other primary dystonias [84]. fMRI and FDG (Fluorodeoxyglucose) PET changes involve the thalamus [83,85]. Increased white matter volume was detected in the subthalamic area that connects the cerebellum and thalamus [86]. A subcortical myoclonus generator is probable, as there is no giant SEP, no back-averaged cortical potential, no abnormal intracortical inhibition, no long-loop reflex pathology and no abnormal cortical excitability in TMS studies [87,88,89,90]. In one small TMS study, active motor threshold was higher than normal, though [91]. Blood flow in the frontemporal cortex and striatum is reduced [92].

Clonazepam, trihexyphenidyl, valproic acid, topiramate or sodium oxybate may be used for symptomatic treatment. Zonisamide has been suggested as a novel treatment option [93,94], and some cases have been reported to respond to tetrabenazine. Substantial improvement after deep brain stimulation in the GPi (internal globus pallidus) or VIM (Ventral intermediate nucleus of the thalamus) has been reported [95], and even patients with isolated myoclonus may benefit from deep brain stimulation in the GPi [96]. Deep brain stimulation might be less effective in mutation-negative myoclonus dystonia [97].

Until the 1980s, several small case series have reported essential myoclonus as an autosomal dominant trait in families without evidence of other clinical symptoms, often responsive to alcohol or clonazepam [98,99,100,101,102,103]. Segmental as well as multifocal myoclonus of the upper body has been described. Without resource to genetic ascertainment, it remains dubious if all these early cases represent part of the spectrum of myoclonus dystonia.

#### 2.2.2. Orthostatic Myoclonus

Orthostatic myoclonus probably is underdiagnosed [104]. The age at onset usually is over 65 years. Palpation or auscultation of the limbs are helpful but unreliable indicators of the presence of orthostatic myoclonus. Thus, only surface polymyography will reliably demonstrate synchronous bursts without stimulus-sensitivity. Gait (stride length, freezing) and balance in affected patients are often abnormal, and difficulties in gait initiation may share features with Parkinson’s disease or normal pressure hydrocephalus [105,106]. One third of patients suffer from a parkinsonian syndrome and one third from severe vascular encephalopathy [104].

Burst duration is about 20–100 ms. In one retrospective case series, the median frequency of arrhythmic bursts of less than 75 ms varied between 5.5 and 12 per second [105]. Other authors have indicated common frequencies between only 3 and 7 per second [104]. Some of the individuals show postural myoclonus of lower frequencies in sitting position [105]. Short symmetric bursts at 9.5–15 Hz in rhythmic epochs in combination with irregular bursts seem to define orthostatic myoclonus in Parkinson’s disease [107], probably not induced by dopaminergic drugs. A frontal subcortical myoclonus generator is conceivable, with possible hyperactivation of anticipatory postural adjustment control mechanisms [104].

Drop attacks might be due to negative orthostatic myoclonus, but this mechanism has not yet been corroborated electrophysiologically. Orthostatic tremor may coexist in some patients [104].

Levetiracetam or clonazepam may be helpful in Parkinson’s disease, sometimes in combination with dopaminergic medication [104,107]. However, beneficial effects are rather small. Many patients find relief in leaning forward and shifting weight onto their arms; e.g., on a walker [104].

#### 2.2.3. Chronic Posthypoxic Myoclonus

In contrast to acute posthypoxic myoclonus, the chronic form (Lance Adams syndrome) is a multifocal action or intention myoclonus that may share reflexive components. It develops in a delayed fashion (days to weeks, sometimes months) after hypoxia, persists over the life-time and is often more disabling than the cognitive consequences of hypoxia. Electrophysiological tests such as back-averaging have made plausible a cortical origin in many cases, although reticular reflex myoclonus is also possible [108]. Pathological structural and functional imaging findings in various cortical or subcortical regions have been associated with chronic posthypoxic myoclonus [69]. Therapeutically, valproic acic, levetiracetam, piracetam or clonazepam may be employed with limited benefits, however.

#### 2.2.4. Cerebrovascular Disease with Probable Subcortical Sources

Brainstem-generated myoclonus seems to underlie cases of myoclonus in basilar occlusion, superficially resembling short generalized seizures in unconscious patients. In single cases, unilateral subcortical infarction was related to generalized myoclonus due to an undefined mechanism [109]. Focal myoclonus has also been reported following thalamic hemorrhage [110]. Other small case series failed to ascertain cases of post-stroke myoclonus [111].

### 2.3. Segmental and Peripheral Myoclonus

Forms of segmental or peripheral myoclonus are relatively rare. Spinal segmental myoclonus refers to myoclonus in spinal muscles of one or several contiguous myotomes of the spinal cord (see below). Propriospinal myoclonus is a peculiar syndrome of slowly propagated movements sparing the face, often with a burst duration incompatible with other types of myoclonus. Its character as a somatic disease has been challenged recently (see below).

Palatal myoclonus is now mainly regarded as a misnomer, has been reclassified as palatal tremor and will therefore not be covered here. Palatal tremor may also represent a psychogenic movement disorder.

Hemifacial spasm is mainly caused by vascular compression of the facial nerve at its exit from the brain stem (65%) or by compression of cerebellopontine angle tumors. Chronic compression seems to predispose to focal ephaptic transmission giving rise to involuntary facial spasms. Less than 3% of cases present with bilateral symptoms [112]. Botulinum toxin relieves symptoms at least partially and often for many months, while carbamazepine or gabapentin is less effective. A resolution of symptoms following microvascular decompression has been reported in about 90% of patients with neurovascular contact over 3 years of follow-up. Vascular decompression has been effective even in rare (2%) familial cases [113].

Hemimasticatory spasm is a similar but rare disorder of the motor branch of the trigeminal nerve which is characterized by unilateral, paroxysmal contractions of the jaw muscles, often triggered by voluntary movement. A loss of the silent period has been reported [114]. Microvascular decompression of the motor branch of the trigeminal nerve was successful in single cases. Rare cases of isolated and reversible facial action myoclonus due to amantadine and bupropion were brought to attention [115]. Other facial myoclonus disorders include postanoxic or serotonin syndrome-related facial myoclonus or blepharoclonus [116]. Hereditary chin trembling might represent a focal variant of essential myoclonus [117].

Hiccups may be regarded as a focal myoclonus. A single case report mentioned hiccups as the unusual main manifestation of an epileptic disorder. As mentioned earlier, hiccups were observed epidemically in the 1920s.

In spinal segmental myoclonus, spinal muscles of one or several contiguous segments of the spinal cord show rhythmical or irregular jerks at rest. The rate is some 1–3/s, and jerks sometimes are stimulus-sensitive. Discharge duration varies between 50–500 ms [16]. It might be difficult to distinguish segmental myoclonus from fasciculations [118], or crural myoclonus-like movements from restless-legs syndrome [119]. Transient segmental myoclonus following herpes zoster infection with recovery in less than 6 months has been reported as a variant of postinfectious myoclonus [120]. Radicular myoclonus induced by neck movement has been described [121]. In segmental myoclonus, treatments such as clonazepam, levetiracetam, tetrabenazine, botulinum toxin injections or intrathecal baclofen have been shown useful in selected cases [122].

### 2.4. Propriospinal Myoclonus

Propriospinal myoclonus (PSM), first described in 1991, has since been increasingly identified as a functional movement disorder in the majority of cases. On the other hand, symptomatic myoclonus has been claimed in about 20% of cases to be due to spinal lesions, neuroinfections, medication or paraneoplastic diseases [123]. Only in single cases, though, the level of spinal abnormalities corresponded with the start of the myoclonic jerks [123,124]. The relevance of spinal cord abnormalities limited to diffusion tensor imaging remains unknown [125].

The onset is often acute in middle age. Jerks may occur spontaneously or stimulus-induced. Myoclonic jerks of 15–5000 ms duration start in the thoracic region and tend to propagate at a rate of 3–15 m/s, producing a repetitive, jerky flexion of the trunk, neck or lower extremities, most often in the supine position [123].

Of 179 cases reported between 1991 and 2014, about 60% have been interpreted as functional myoclonus [126]. Similar movements may be produced willfully. Propriospinal pathways in the human spinal cord have remained anatomically ill-defined [124]. In a monocentric series of patients with “axial jerks”, 34 of 35 patients were judged to have a psychogenic movement disorder [127] on the basis of the presence of psychiatric comorbidity, a Bereitschaftspotential preceding the jerks or inconsistent EMG characteristics (differences in initial muscle, highly variable pattern of muscle activation). A phenomenological overlap with adult motor tics has been suggested [123,127]. Long-latency abdominal reflexes showed variable latencies [128].

Characteristics of functional movement disorders include spontaneous variability of presentation, entrainment of frequency by other motor tasks, distractability, coactivation of agonist and antagonist muscles or “bizarre” posturing, among others, but none of these features in isolation differentiates reliably from organic movement disorders [129]. Pre-movement potentials (Bereitschaftspotential, BP; 1965) over the contralateral pre-motor cortex precede willful movements, but double dissociation has been recorded in functional disorders. There have been reports of involuntary movements preceded by a slow negative EEG shift, as well as reports of normal subjects with no BP before voluntary movements [130]. BP has not been found in 35–50% of cases classified as functional disorders [123,127].

The following definition of idiopathic PSM has been proposed [123]: a start in thoracic muscles while lying down and during relaxation; simultaneous, invariant propagation up and down the spinal cord at low velocity; reliable stimulus sensitivity; and absence of a BP. One might add strict rhythmicity, burst duration below 75 ms and persistence during sleep as criteria unlikely for a psychogenic disorder.

Symptomatic cases might respond to clonazepam or botulinum toxin. Only half of the patients with psychogenic movement disorders improve with multimodal therapy during long-term follow up [131].

## 3. Negative Myoclonus

Physiological negative myoclonus may appear during fear or sleep transition. Negative myoclonus consists of irregular lapses of muscle activity and posture. Pathological negative myoclonus can be tested by extending the arms and dorsiflexing the wrists, or at the hips in a supine position with knees bent and feet resting on the bed, leaving the legs to fall to the sides [132]. Negative myoclonus can also produce a wobbling gait or sudden postural lapses (“bouncy gait”), for example after cerebral hypoxia [5].

Asterixis as its most characteristic subtype is associated with repetitive, semi-rhythmic pauses of 25–200 ms in EMG activity, interrupting tonic muscle contraction. They might be preceded by short EMG bursts, e.g., in posthypoxic myoclonus, in which case shorter burst durations imply generators positioned nearer to the cortex [18]. Asterixis has classically been described in the context of metabolic encephalopathy; e.g., as a flapping tremor in hepatic encephalopathy. However, asterixis may also occur in various drug-induced disorders; in particular, some antiepileptic drugs, cerebral malaria, Huntington’s disease, Rolandic epilepsy or generalized epilepsies, among others. It may also make a transient appearance in the elderly [133].

Some 20% occur unilaterally, most often due to focal cerebrovascular disease [134]. More than half of the focal lesions seem to involve the thalamus [135], but lesions of the contralateral sensorimotor cortex, internal capsule, brainstem or the ipsilateral cerebellum have also been associated.

Frontoparietal cortical (pre or post-central cortex, premotor cortex and supplementary motor area) as well as subcortical generation of myoclonus is conceivable. When cortical negative myoclonus is encountered, positive myoclonus is often also present, and silent periods occur bilaterally non-synchronously, less rhythmically and are more stimulus-sensitive than with the subcortical origin of negative myoclonus [17]. In cortical negative myoclonus, associated giant SEPs or a time-locked EEG transient 25–50 ms prior to the muscular silent period has been detected [136,137]. There seem to exist areas of the primary sensorimotor cortex that, when stimulated electrically of magnetically, produce silent periods in dependent muscles, either by activation of inhibitory cortical areas or by activation of spinal inhibitory interneurons [15]. Negative myoclonus can be generated by low-level electric stimulation of the premotor cortex, motor cortex, somatosensory cortex, or by the level-independent stimulation of the supplementary motor area [138]. Postcentral spikes detected by subdural electrodes suggested a postcentral generator of negative myoclonus in an epilepsy patient, for example [139]. Pontomedullar centers can inhibit spinal motor neurons [17].

There is usually less treatment effect from antimyoclonic drugs in negative myoclonus compared to positive myoclonus, although good responses to levetiracetam have been reported. Carbamazepine, phenytoin, as well as other antiepileptic drugs should be avoided as these may lead to or worsen negative myoclonus.

## 4. Metabolic Causes of Myoclonus

Myoclonus and tremor probably are the most frequent movement disorders in the intensive care unit, frequently as part of an encephalopathic syndrome. The identification of the underlying abnormalities (often multiple) in critically ill patients might be particularly challenging [71]. Encephalopathies are characterized by diffuse and bilateral brain dysfunction, often on a metabolic, toxic or inflammatory basis. The onset is often acute or subacute and brain dysfunction may be reversible, once triggers (often multiple) have been eliminated. Apart from disturbances in consciousness, cognition, drive or emotion, there may be non-specific features such as dizziness, nausea, headaches, seizures or autonomic symptoms. In many encephalopathies, positive or negative myoclonus represents a prominent finding. Among them are hyponatremia, hepatic encephalopathy, uremia, hypophosphatemia, hypo or hyperglycemia, septic encephalopathy, pulmonary encephalopathy (carbon dioxide retention), hyperthyroidism or steroid-responsive encephalopathy with autoimmune thyroid disease (SREAT) [140].

### 4.1. Uremic Encephalopathy

Uremic encephalopathy may affect up to 20% of patients whose kidney function has deteriorated rapidly (GFR < 20 mL/min) to a degree where dialysis becomes necessary. Following the introduction of dialysis, full-blown uremic encephalopathy has become rare and reversible. General weakness, dysarthria, incoordination, chorea, action tremor or rigidity may be symptoms that accompany positive or negative myoclonus (asterixis). Myoclonic jerks may occur action-related or stimulus-responsive [140]. Non-specific EEG changes such as general slowing, frontal intermittent delta activity, delta bursts or triphasic waves may be encountered. Neurotoxic guanidino compounds that affect NMDA (N-Methyl-D-Aspartate), glycine or GABA transmission have been implicated in pathophysiology of uremia. Dialysis disequilibrium syndrome with myoclonus has become rare.

Action myoclonus renal failure syndrome presents as a rare inherited disease in the second or third decade. SCARB2 (scavenger receptor class B member 2 protein) gene mutations encoding lysosomal integral membrane protein type 2 (LIMP2) are responsible (Table 2) [25].

### 4.2. Hepatic Encephalopathy

Hepatic encephalopathy (HE) affects 10–15% of liver cirrhosis patients, and its cumulative incidence in liver cirrhosis reaches 30–40%. Disorientation or asterixis often marks the onset of overt HE [141], and asterixis may be encountered in grade I to III HE. Ammonia levels can be normal in 10–20% of patients and do not correlate with the severity of encephalopathy, except for acute HE. MRI in liver cirrhosis may show T2 hyperintense lesions in pallidum, striatum or substantia nigra, but this is independent from the presence of HE. EEG changes include general slowing, triphasic waves or sharp waves. Therapy consists of the elimination of triggers and lactulose (or lactitol), which may be combined with rifaximin after relapse. l-ornithin-l-aspartate or branched amino acids may be complementary or alternative treatment strategies.

Rare inherited metabolic diseases with myoclonus include glutaraciduria, Biotin-responsive biotinidase deficiency, galactosialidosis or sialidosis [142].

## 5. Autoimmune Conditions with Myoclonus

### 5.1. Opsoclonus-Myoclonus Syndrome

Opsoclonus-myoclonus syndrome (OMS) occurs as a para or post-infectious, paraneoplastic or autoimmune disease on a neuroinflammatory basis. Ataxia is the third prominent feature of a triad involving the eponymous opsoclonus and myoclonus. Opsoclonus describes involuntary, rapid, nonrhythmic horizontal and vertical oscillations of the eyes. In adult patients, myoclonus involves the extremities more frequently than the craniocervical region or trunk [143]. In 41 children with OMS due to neuroblastoma, 68% had myoclonus, but just 59% showed the complete triad of opsoclonus, myoclonus and ataxia [144]. Myoclonus duration is below 100 ms, and back-averaging, EEG studies, SEP amplitudes and long-latency responses have been found to be normal [145].

Infectious contexts include Lyme disease, VZV (Varizella zoster virus), EBV (Epstein Barr virus), Coxsackievirus, West Nile virus or streptococcal infection. In autoimmune cases, antibodies against the NMDA receptor, AMPA/GluR3 receptor, mGluR5 receptor, GABA-B-receptor, LGl1 or GAD may be detected. In a paraneoplastic setting in children, neuroblastomas can most often be found, while in adults small cell lung cancer or breast cancer, rarely melanomas or non-Hodgkin lymphomas, are associated [143,146]. Rarely, Ri or Hu antibodies have been identified in paraneoplastic cases.

Monophasic, relapsing-remitting or chronic relapsing disease patterns may be observed, while the latter type predominates in children (60%) [147].

Corticosteroids, intravenous immunoglobulins, cyclophosphamide, azathioprine, rituximab or plasmapheresis have been applied to mitigate the supposedly autoimmune disease process [148,149]. In children, combination therapy with corticotrophin seems to improve treatment response [150]. Importantly, in some cases treatment of the underlying cancer improves OMS. Following treatment, remission can be observed in two thirds of patients. Nevertheless, long-term deficits in motor performance and speech affect two thirds of pediatric patients, and half of them suffer from long-standing learning or behavioral problems [147].

### 5.2. Steroid-Responsive Encephalopathy with Autoimmune Thyroid Disease

Steroid-responsive encephalopathy with autoimmune thyroid disease (SREAT) is a contentious disease entity distinguished by confusion, cognitive disorders or reduced level of consciousness (40–50%), epileptic seizures (50%), myoclonus (35%) and tremor (30%). Importantly, normal thyroid function is found in 75% [151]. Thyroid peroxidase (TPO) antibodies in serum are deemed obligatory for diagnosis and they are also present in cerebrospinal fluid in 60–80% of cases. Furthermore, thyreoglobulin antibodies (70%) or α-enolase antibodies (50%) may be detected. On the other hand, 10–20% of healthy individuals have TPO antibodies in serum, and antibody levels do not correlate with the course of the disease. Currently, TPO antibodies are relegated to the status of a mere marker of autoimmune susceptibility. Cerebrospinal fluid may show elevated protein (60–85%), oligoclonal bands (30%) and rarely pleocytosis (<20%). Focal or diffuse white matter changes in MRI are identified in 10–50% and may be reversible. Steroid responsiveness after days to weeks has been included in disease definition but may be incomplete. In this case, azathioprine, cyclophosphamide, intravenous immunoglobulins or plasmapheresis have been used.

### 5.3. Stiff-Person Syndrome and Progressive Encephalomyelitis with Rigidity

Progressive encephalomyelitis with rigidity (PERM) is an autoimmune encephalopathy, which may become manifest with dementia, hallucinations, brainstem signs, autonomic disturbances, myoclonus (25%) or an akinetic-rigid syndrome. As with OMS, autoimmune cases (with antibodies to GAD, amphiphysine, DPPX or the glycine receptor) coexist with paraneoplastic cases due to thymoma, breast or lung cancer [152,153,154]. Diazepam, methylprednisolone, intravenous immunoglobulins, plasmapheresis or rituximab are therapeutic options. Stiff-person syndrome is a similar, more restricted disease involving muscular hypertonia, muscular spasms and psychiatric comorbidities.

### 5.4. Limbic Encephalitis

In cases of limbic encephalitis with LGl1 antibodies, distinctive facio-brachial motor phenomena may be observed that correspond to focal seizures. LGl1 interacts with a presynactic and postsynaptic receptor to form a trans-synaptic complex that includes potassium channels and the AMPA receptor. In CASPR2 antibody-positive limbic encephalitis, myoclonus may also rarely occur.

## 6. Infectious und Para-Infectious Causes of Myoclonus

Several neuroinfections might involve myoclonus. In genuine encephalitis, myoclonus is often accompanied by alterations in mental state and seizures. On the other hand, para-infectious myoclonus might be misinterpreted as epilepsy in affected children [155]. Historically, a flare of myoclonic encephalitis and epidemic hiccups formed part of the wave of encephalitis lethargica in the years following the first world war [156]. Different types of myoclonus have been recorded in HIV infection [157]. In Whipple’s disease, oculomasticatory myorhythmia, sometimes involving myorhythmia of skeletal muscles, can be encountered less frequently (20%) than non-rhythmic myoclonus (25%) [158]. Myoclonic jerks might also be induced by viral (VZV, HSV-1, EBV, measles, mumps, WNV, St. Louis encephalitis, EEE (Eastern equine encephalitis) virus, WEE (Western equine encephalitis) virus, Dengue virus), bacterial (Leptospira, *Mycoplasma pneumonia*), parasitic (cerebral malaria) or slow virus infections (subacute sclerosing panencephalitis) [159].

## 7. Medication and Toxin-Induced Myoclonus

A minimum incidence of 0.2% of drug-induced myoclonus has been suggested in a French study [160]. Most frequently opioids, antidepressants, classic or atypical antipsychotics, antiepileptic drugs or antibiotics have been reported to underlie reversible medication-induced myoclonus [161]. Similarly, multiple agents can induce myoclonus in animal models of myoclonus. Several neurotransmitter pathways seem to be involved in drug-induced myoclonus, including the neurotransmitters glutamate, glycine, GABA, dopamine and serotonin.

Among antibiotic subgroups, beta-lactams, quinolones, sulfonamides or aminoglycosides can be found responsible most frequently. Furthermore, levodopa, dopamine agonists, anticonvulsant drugs, non-steroidal anti-inflammatory drugs, bismuth salts, lithium, amantadine, memantine, metoclopramide, calcium antagonists, corticosteroids, opioids, contrast media, chemotherapeutic drugs, propofol, as well as chronic intoxication with toluene, gasoline sniffing or substance withdrawal may be causally related [25,71,159,161,162]. With long-time antipsychotic use, a rare tardive myoclonus may occur. In some cases, an EEG transient related to the myoclonic jerks has suggested a cortical origin of drug-induced myoclonus.

As an important differential diagnosis, the serotonin syndrome results from the inadvertent combination of serotonergic drugs and may show hyperreflexia, autonomic disturbance, dramatic agitation and other mental changes besides myoclonus. In most cases, discontinuation will lead to symptom relief.

## 8. Transient Myoclonus of the Elderly—A Specific Entity?

Myoclonus of the face, neck and upper extremities at rest, sometimes aggravated by posture or action, has been reported as a transient phenomenon in elderly patients without identifiable causes [163]. Positive as well as negative myoclonus has been observed [133]. It might be suspected that dehydration, metabolic and drug-induced factors co-operate in the generation of myoclonus in these cases. Clonazepam has been found to be effective [163].

Less favorable is the prognosis in individuals over 65 years with progressive asymmetric action myoclonus of unknown cause (primary progressive myoclonus of aging) [164].

An intriguing case-report of “cyclic” myoclonus and fever of unknown etiology, appearing every three days and aborted by benztropine, has been presented [165].

## 9. Conclusions

It can be a challenging task to distinguish cortical myoclonus from myoclonus with non-cortical sources, but this distinction might help guide initial therapy. Electrodiagnostic tests still play a prominent part in making this distinction. Cortical myoclonus is a condition typically characterized by abnormally enlarged somatosensory evoked potentials, exaggerated long latency reflexes (LLR) and a pre-myoclonic spike recorded with jerk-locked back-averaging [14]. Abnormal long latency reflexes may also be found in subcortical reflex myoclonus. Cortical generators of myoclonus have been challenged for many disorders with generalized EEG discharges, SEP and LLR studies, however, and a case has been made for a subcortical contribution to myoclonogenesis in these cases [9]. Although the differential diagnosis of myoclonus remains broad even after subtyping myoclonus, helpful and pragmatic approaches have been suggested in recent years that narrow the list of diagnoses to be considered [159,166].

The genetic dissection of myoclonus has made rapid and important advances in the past few years, particularly regarding genetic causes of epileptic myoclonus and of neurodegenerative diseases with myoclonic features. The search for yet undetected genetic sources of myoclonus has accelerated with the advent of next-generation sequencing and will certainly help to deepen our understanding of the different pathophysiologies of myoclonus. It is obvious that the anatomical basis of myoclonus is more diverse than previously thought, and that alterations in multiple neurotransmitter pathways may contribute to myoclonus, particularly of those that enhance cortical excitability. Pathophysiology may be different for some forms of negative myoclonus, in which short periods of cortical silencing can be observed. Some types of myoclonus have puzzlingly joined the ranks of psychogenic movement disorder; e.g., the majority of cases of propriospinal myoclonus, or some cases of palatal tremor previously classified as palatal myoclonus. For practical purposes, a very thorough approach in history taking, including family history, careful consideration of potential causes of myoclonus is mandatory. Given the heterogeneity of genetic causes and the fact that genetic testing frequently is not available as simple routine test, search for mutations might prove most helpful if clinical data suggest a phenotype such as myoclonus-dystonia.

Useful summaries of current treatment approaches for myoclonus have been published [10,16,122,131]. Still, randomized controlled trials are scarce for any type of myoclonus [131]. For cortical myoclonus, levetiracetam, valproic acid, clonazepam or piracetam may be effective, among others. In many types of subcortical myoclonus, clonazepam is the drug of choice, but diminishing effects due to tolerance may ensue. Deep brain stimulation renders satisfying therapeutic effects in myoclonus dystonia.

Disorders with particular relevance to the field of myoclonus and with known mutations or known genetic loci are mentioned. This is not meant to be an exhaustive list of all genetic disorders in which myoclonus may appear. The number of known mutations is increasing steadily.

## Figures and Tables

**Table 1:** Different approaches at the classification of myoclonus.

Distribution of Jerks	Mode of Presentation	Anatomical Source	Etiology
focal segmental axial multifocal generalized	rest action stimulus-induced (sensory, visual, auditory, emotional)	cortical cortical-subcortical subcortical segmental (peripheral)	physiologic essential epileptic symptomatic functional

**Table 2:** Genetic dissection of myoclonic disorders.

Disease	OMIM Phenotype Number	Age of Onset	Selected Additional Clinical Features	Mode of Trans-Mission	OMIM Gene/Locus Number	Mutated Gene or Genetic Loci
**Hyperekplexia Syndromes**						
Hereditary hyperekplexia-1 (HKPX1)	149400	<1 year	Exaggerated startle response, seizures	AR, AD	138491	GLRA1
Hereditary hyperekplexia-2 (HKPX2)	616619	<1 year	Exaggerated startle response, seizures	AR	138492	GLRB
Hereditary hyperekplexia-3 (HKPX3)	614618	<1 year	Exaggerated startle response	AR, AD	604159	SLC6A5
**Myoclonic Epilepsies**						
Juvenile myoclonic epilepsy (EJM 1–9)	1: 254770 2: 604827 3: 608816 4: 611364 5: 611136 6: 607682 7: 613060 8: 607628 9: 614280	8–20 years	Seizures, normal intelligence	AD	1: 608815 5: 137160 6: 601949 7: 137163 8: 600570	1: EFHC1 2: 15q14 3: 6p21 4: 5q12–q14 5: GABRA1 6: CACNB4 7: GABRD 8: CLCN2 9: 2q33–q36
Unverricht–Lundborg disease (EPM1A)	254800	6–16 years	Ataxia, dystonia, dementia	AR	601145	Cystatin B
EPM 1B	612437	5–10 years	Ataxia	AR	608500	PRICKLE1
Lafora disease (EPM2A/B)	254780	8–18 years	Dementia, visual disturbance	AR AR	A: 607566 B: 608072	A: EPM2A B: NHLRCI
EPM3/CLN14	611726	<2 years	Developmental regression	AR	611725	KCTD7
EPM4/action myoclonus-renal failure syndrome (AMRF)	254900	14–30 years	Renal failure (most)	AR	602257	SCARB2
EPM6	614018	1–3 years	Ataxia, sensory neuropathy	AR	604027	GOSR2
EPM7/myoclonus epilepsy and ataxia due to potassium channel mutation (MEAK)	616187	6–14 years	Cognitive decline (some)	AD	176258	KCNC1
EPM8	616230	6–16 years	Dementia	AR	606919	CERS1
EPM9	616540	6–7 years	Gait disturbance	AR	150341	LMNB2
EPM10	616640	<10 years	Spastic ataxia, cognitive decline	AR	616639	PRMD8
Neuronal ceroid lipofuscinoses (NCL)	1: 256730 2: 204500 3: 204200 4A: 204300 4B: 162350 5: 256731 6: 601780 7: 610951 8: 600143 9: 609055 10: 610127 11: 614706 12: 606693 13: 615362 14: 611726	Variable (infantile, juvenile, adult forms)	Mental retardation/dementia, seizures, ataxia, visual failure	AR 4B: AD	1: 600722 2: 607998 3: 607042 4A: 606,725 4B: 611,203 5: - 6: 602780 7: 611124 8: 607837 9: ? 10: 116840 11: 138945 12: 610513 13: 603539 14: 611725	CLN1: PPT1 CLN2: TPP1 CLN3: Battenin CLN4A: CLN6 CLN4B: DNAJC5 CLN5: CLN5 CLN6: CLN6 CLN7: MFSD8 CLN8: CLN8 CLN9: ? CLN10: cathepsin D CLN11: GRN CLN12: ATP13A2 CLN13: CTSF CLN14: KCTD7
Sialidosis type I/II	256550	0.5–14 years	Seizures, ataxia, low IQ, visual failure	AR	608272	NEU1
Severe myoclonic infantile epilepsy (SMEI/Dravet syndrome)	607208	<1 year	Mental retardation	AD	182389 603415	SCN1A (80%) SCN9A
Neonatal intractable myoclonus (NEIMY)	617235	At birth	Seizures, chorea, developmental arrest, dysphagia	AD	602821	KIF5A
Familial infantile myoclonic epilepsy (FIME)	605021	<1 year	Seizures	AR	613577	TBC1D24
Myoclonic-atonic epilepsy (MAE)	616421	<3 years	Intellectual disability, seizures	AD	137165	SLC6A1
Spinal muscular atrophy with Progressive myoclonic epilepsy (SMA-PME)	159950	2–12 years	Weakness and atrophy, dysphagia	AR	613468	ASAH1
Angelman syndrome	105830	<1 year	Mental retardation, aphasia, microcephaly, ataxia	Maternal imprinting (some)	601623	del15q11.2–q13 Ubiquitin E3 ligase (UBE3A)
Gaucher disease type III	231000	0.5–50 years	Gaze palsy, seizures, dementia, spasticity, parkinsonism	AR	606463	GBA
Niemann–Pick disease type C	257220	1–55 years	Dementia, seizures, spasticity, ataxia, speech disturbance	AR	607623	NPC1
GM2 gangliosidosis type I/II/AB variant	272800 268800 272750	Most <1 year, rare adult onset	Blindness (cherry red spot), spasticity, dementia, seizures	AR	I: 606869 II: 606873 AB: 613109	I: HEXA II: HEXB AB variant: GM2A
Glucose transporter 1 deficiency (GLUT1DS1)	606777	<1 year	Seizures, ataxia, spasticity, mental retardation	AR, AD	138140	SLC2A1
Benign adult familial myoclonic epilepsy 1–5 (BAFME or FAME)	1: 601068 2: 607876 3: 613608 4: 615127 5: 615400	Mean 37 years, some childhood	Seizures	AD	2: 104260 5: 190197	1: 8q24 2: ADRA2B 3: 5p15 4: 3q26.32–q28 5: CNTN2
Myoclonus epilepsy with ragged-red fibers (MERRF)	545000	6–48 years	Myopathy, hearing loss, ataxia, spasticity	mitochondrial	590060 590050 590040 590080 590085 590070 et al.	MTTK, MTTL1, MTTH, MTTS1, MTTS2, MTTF et al.
**Neurodevelopmental Disorders, Movement Disorders and Neurodegenerative Diseases with Myoclonus**						
Alpers syndrome	203700 607459	Alpers: <3 years	Liver failure, ataxia, seizures, dementia	AR	174763	POLG depletion or mutation
Cerebro-tendinous xanthomatosis (CTX)	213700	12–44 years	Mental retardation/dementia, spasticity, pseudobulbar palsy	AR	606530	CYP27A1
Spinocerebellar ataxia (SCA) with epilepsy (formerly EPM5)	607459	5–17 years	Ataxia, ophthalmoplegia	AR	174763	POLG
Wilson disease	277900	5–40 years	Liver cirrhosis, tremor, dysarthria, dementia, dystonia	AR	606882	ATP7B
Familial Parkinson’s disease (PARK1, PARK4)	168601 605543	16- >60 years	Parkinsonism, dementia	AD	163890	α-synuclein mutation, α-synuclein triplication
Spinocerebellar ataxia 2 (SCA2)	183090	2–65 years	Cerebellar ataxia, dysphagia, dementia, parkinsonism, polyneuropathy	AD	601517	ATXN2
Spinocerebellar ataxia 13 (SCA13)	605259	4–60 years	Cerebellar ataxia, pyramidal signs	AD	176264	KCNC3
Dentatorubral-pallidoluysian atrophy (DRPLA)	125370	0.5–30 years	Dementia, ataxia, choreoathetosis	AD	607462	ATN1
Huntington’s disease (HD)	143100	2- >60 years	Chorea, dementia, parkinsonism, seizures (juvenile)	AD	613004	HTT
Familial Alzheimer disease 3	607822	26–62 years	Dementia, dysarthria, dystonia, spasticity, aphasia	AD	104311	PSEN1
Creutzfeldt–Jakob disease (CJD), Gerstmann–Sträussler–Scheinker disease (GSD), familial fatal insomnia (FFI)	123400 137440 600072	Mean 52 years GSD: 38–70 years	Dementia, ataxia, parkinsonism, aphasia	AD	176640	PRNP
Familial cortical myoclonus (FCM)	614937	10–70 years	Ataxia	AD	605235	NOL3
**Myoclonic Dystonias**						
Myoclonic dystonia (DYT11)	159900	1–18 years	Dystonia, tremor, psychiatric comorbidity	AD	604149	SGCE
Myoclonic dystonia (DYT15)	607488	5–15 years	Dystonia	AD	-	18p11
Myoclonic dystonia (DYT26)	616398	<20 years	Dystonia	AD	616386	KCTD17

OMIM: Online Mendelian Inheritance in Man.

## Abbreviations

List of abbreviations used in the main text

AD	autosomal dominant
AMPA	α-amino-3-hydroxy-5-methyl-4-isoxazolepropionic acid
APP	amyloid precursor protein
AR	autosomal recessive
ATP7B	ATPase copper transporting beta
BAFME2	benign adult familial myoclonic epilepsy 2
BP	Bereitschaftspotential
CJD	Creutzfeldt-Jakob disease
DNA	desoxyribonucleic acid
DPPX	dipeptidyl-peptidase-like protein 6
DRPLA	dentatorubral-pallidoluysian atrophy
EBV	Epstein Barr virus
EEE	Eastern equine encephalitis
EEG	electroencephalography
EMG	electromyography
EPM	progressive myoclonic epilepsy
FDG	fluorodeoxyglucose
fMRI	functional magnetic resonance tomography
GABA	gamma-aminobutyric acid
GAD	glutamic decarboxylase
GFR	glomerular filtration rate
GluR	glutamate receptor
GM2	monosialic ganglioside 2
GPi	globus pallidus internus
GTP-CH	guanosine triphosphate cyclohydrolase
HE	hepatic encephalopathy
Hz	Hertz
HSV-1	herpes simplex virus 1
JME	juvenile myoclonic epilepsy
LGl1	anti-leucine-rich glioma inactivated-1
LHON	Leber hereditary optic neuropathy
LIMP 2	lysosomal integral membrane protein type 2
LLR	long latency reflexes
m	meter
MEG	magnetoencephalography
MELAS	mitochondrial encephalomyopathy, lactic acidosis, and stroke-like episodes
MERFF	myoclonic epilepsy with ragged red fibers
mGluR	metabotropic glutamate receptor
MRS	magnetic resonance spectroscopy
ms	millisecond
NMDA	N-methyl-D-aspartate
OMS	opsoclonus-myoclonus syndrome
PERM	progressive encephalomyelitis with rigidity
PET	positron emission tomography
PME	progressive myoclonic epilepsies
POLG	DNA polymerase gamma
PSM	propriospinal myoclonus
s	second
SCA	spinocerebellar ataxia
SCARB2	scavenger receptor class B member 2
SGCE	sarcoglycan-ε
SEP	somatosensory evoked potentials
SREAT	steroid-responsive encephalopathy with autoimmune thyroid disease
TMS	transcranial magnetic stimulation
TPO	thyroid peroxidase
VIM	ventral intermediate
VZV	varizella zoster virus
WNV	West Nile virus
WEE	Western equine encephalitis
µV	microvolt
